# Production and Characterization of a Bioemulsifier Derived from Microorganisms with Potential Application in the Food Industry

**DOI:** 10.3390/life12060924

**Published:** 2022-06-20

**Authors:** Jaffar Z. Thraeib, Ammar B. Altemimi, Alaa Jabbar Abd Al-Manhel, Tarek Gamal Abedelmaksoud, Ahmed Ali Abd El-Maksoud, Chandu S. Madankar, Francesco Cacciola

**Affiliations:** 1 Department of Food Science, College of Agriculture, University of Basrah, Basrah 61004, Iraq; agripg.jaafar.zamel@uobasrah.edu.iq (J.Z.T.); alaa.abd@uobasrah.edu.iq (A.J.A.A.-M.); 2 Food Science Department, Faculty of Agriculture, Cairo University, Giza 12613, Egypt; tareekgamal_88@agr.cu.edu.eg; 3 Dairy Science Department, Faculty of Agriculture, Cairo University, Giza 12613, Egypt; ahmed_ali@cu.edu.eg; 4 Department of Oils, Oleochemicals and Surfactants Technology, Institute of Chemical Technology, Mumbai 400019, India; chandumadankar@gmail.com; 5 Department of Biomedical, Dental, Morphological and Functional Imaging Sciences, University of Messina, 98125 Messina, Italy

**Keywords:** emulsifiers, food, microbial surfactants, biodegradable, non-toxic, fungi

## Abstract

There is a growing interest in the development and use of natural emulsifiers, which provide biodegradability as well as non-toxicity along with giving better performance compared to existing emulsifying agents used in the food industry. A large variety of sources of starting material, i.e., the microorganisms, are available to be used, hence giving a diverse range of applications. The focus of this review paper is on the production of bioemulsifiers, which are said to be “green surfactants”, from fungi, bacteria and yeasts; furthermore, an overview pertaining to the knowledge gained over the years in terms of characterization techniques is reported. The methods used for the characterization and isolation such as TLC, GC-MS, HPLC, NMR have also been studied. The end-application products such as cookies, muffins, and doughs along with the methods used for the incorporation of bioemulsifiers, microorganisms from which they are derived, properties imparted to the product with the use of a particular bioemulsifier and comparison with the existing food grade emulsifiers has been discussed in detail. The future prospects indicate that newer bioemulsifiers with anti-microbial, anti-oxidant and stabilization properties will prove to have a larger impact, and emphasis will be on improving the performance at an economically viable methodology.

## 1. Introduction

Bioemulsifiers have a larger molecular weight than biosurfactants, because they are complex mixes of lipopolysaccharides, lipoproteins, heteropolysaccharides, and proteins [1]. Due to their functional capabilities and eco-friendly properties, bioemulsifiers (BE) are regarded as multifunctional biomolecules of the twenty-first century [2]. Numerous microorganisms produce bioemulsifiers under a variety of diverse and extreme environmental conditions [3]. Bioemulsifiers are widely used in a variety of industries, including medicine, petroleum, food, pharmaceuticals, chemicals, textiles, and cosmetics [4]. Currently, bioemulsifiers are also referred to as “green molecules” due to their widespread use in soil bioremediation [5]. Their importance in global markets has been growing daily, as they are natural resources with a high aggregate value [6]. Emulsifiers exhibit dual lipophilicity and hydrophilicity. Emulsions are either oil-in-water (O/W) or water-in-oil (W/O) [7]. In O/W emulsions, the dispersed phase consists of discrete small droplets of oil in water, whereas in W/O emulsions, the dispersed phase consists of discrete small droplets of water in oil [8]. Several of these bioemulsifiers have been licensed by the International Organization for Animal Health, including the WHO (World Health Organization); however, the majority of these compounds have been studied nutritionally [9]. Numerous biomolecules are also utilized in the oil, food, pharmaceutical, and chemical industries [10]. Emulsifiers are substances that improve the consistency of fat-soluble vitamins, fatty acids, and amino acids. Emulsions’ function is inextricably linked to their chemical structure [11].

Today, due to the emulsifier’s beneficial effect on human health, scarcity of resources, and high cost, researchers have developed emulsifiers using natural resources, particularly microorganisms. Natural surfactants are referred to as bioemulsifiers because they are derived from biological entities, particularly microorganisms. Numerous species and strains of fungi, bacteria, and yeast are known to produce bioemulsifiers possessing different molecular structures [12]. Microorganisms that produce bioemulsifiers can be classified into three categories [13]: those that produce bioemulsifiers exclusively from alkanes, such as *Corynebacterium* sp.; those that produce biosurfactants exclusively from water-soluble substrates, such as *Bacillus* sp.; and those that produce biosurfactants from both alkanes and water-soluble substrates, such as *Pseudomonas*. The production of emulsifying agents from yeast typically requires the presence of water-insoluble substrates, which complicates the isolation of the bioemulsifiers produced. Ribeiro et al. [14] evaluated the use of bioemulsifiers produced by *Saccharomyces cerevisiae* URM 6670 as a substitute for egg yolk in a cookie formulation. After baking, the bioemulsifiers had no effect on the physical or physicochemical properties of the product. Yeasts produce a variety of emulsifiers, which are particularly interesting given that several yeasts are food-grade, allowing for use in food-related industries. Liposan is an emulsifier produced by *Candida lipolytica* on an extracellular level [15]. Saccharomyces cerevisiae produces mannanprotein emulsifiers. Numerous bioemulsifiers have found applications in the food, cosmetics, and petroleum industries [15].

**Figure 1:**
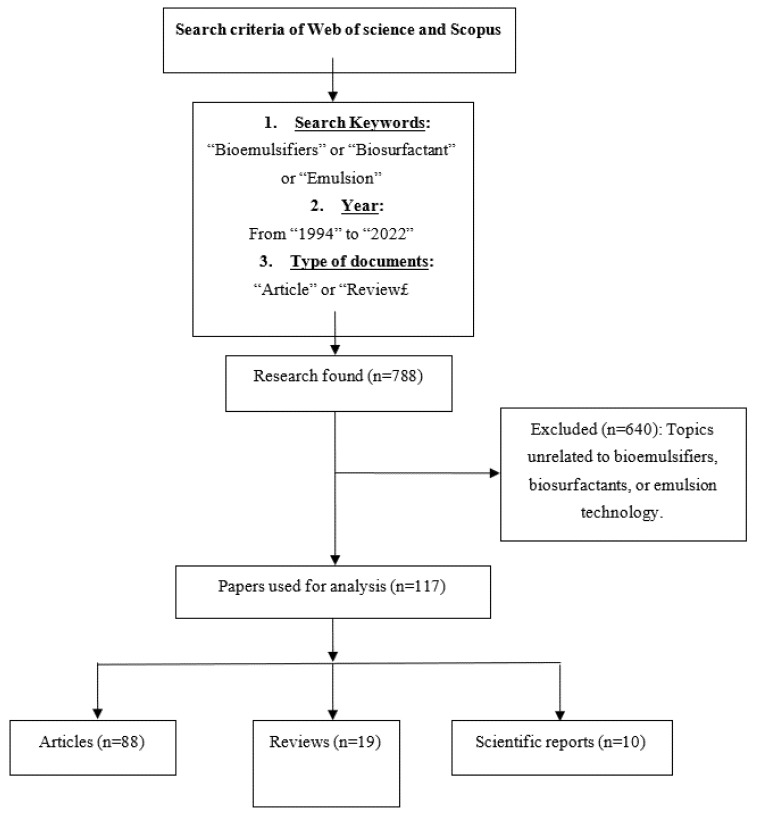
Total of 117 research/review articles referred in paper by searching keywords “Bioemulsifiers” or “Biosurfactants” or “Emulsion” in the Web of Science and Scopus.

The economics of bioemulsifiers production can be significantly reduced by utilizing renewable and low-cost nutrients, e.g., agricultural waste. The optimization of the manufacturing process through identification of the optimal growth medium components and optimal cultivation conditions enables the use of bioemulsifiers with emulsifying capacity in a variety of industries. The search for literature in the Web of Science database was conducted using the keywords “Bioemulsifiers” or “Biosurfactants” or “Emulsion”, and 117 research and review articles were identified for this review (Figure 1). The main goal of the present study is to have a detailed overview of the knowledge gained over the years regarding bioemulsifiers, including the factors influencing its production from microorganism, physicochemical properties, advancements in the incorporation of biomolecules into various industries, and future research needs.

## 2. Bioemulsifiers

Emulsifiers can be synthesized chemically or via microbial metabolism (bioemulsifiers). Bioemulsifiers are versatile chemical compounds that are capable of stabilizing oil-in-water emulsions and are critical in a variety of industrial applications [16]. They are also referred to as biopolymers or polysaccharides with a high molecular weight. Even at low concentrations, these molecules emulsify two immiscible liquids efficiently but are less effective at reducing surface tension. Combining polysaccharides, fatty acids, and protein components in bioemulsifiers enhances their emulsifying capacity [17]. Liposan, produced by *Candida lipolytica*, is the most studied bioemulsifier [18]. It is roughly 17% protein and 83% carbohydrate (polysaccharide–protein complex). The carbohydrate portion contains glucose, galactose, galactosamine, and galacturonic acid.

Emulsan is an extracellular heteropolysaccharide composed of two biopolymers: 20% exopolysaccharide and 80% lipopolysaccharide with a high molecular weight. It was extracted in the late 1970s from a hydrocarbon-degrading Arthrobacter sp. RAG-1 (later renamed *Acinetobacter venetianus* RAG-1) [19]. Emulsan addition improved the stability of alginate microspheres, allowing for the fine-tuning of biological molecule release by using different emulsan concentrations. The authors concluded that emulsan is an excellent candidate for protein and pharmaceutical delivery. Specific emulsan–alginate formulations have been granted patents as medication delivery methods and vehicles for the removal of protein-based toxins from food and/or other items [20,21]. *Acinetobacter radioresistens* was successfully used by Navon-Venezia et al. to produce Alasan [22]. Alasan is a compound of covalently bonded anionic polysaccharides that contain alanine-rich proteins. The emulsifying and surface activities of Alasan have been related to the compound’s three main proteins, which have molecular weights of 16, 31, and 45 kDa. According to Toren et al., the protein with a molecular mass of 45 kDa exhibited the highest emulsifying activity, exceeding even the intact alasan complex [23].

**Figure 2:**
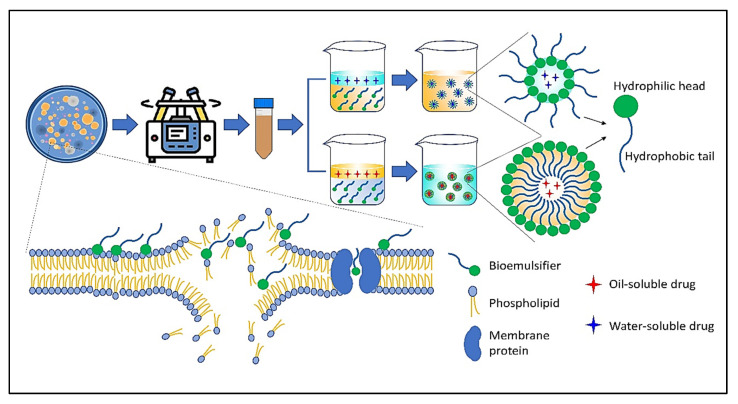
The schematic and action mechanism of bioemulsifiers in emulsion systems.

Mannoproteins are a class of glycoproteins isolated from the cell walls of a variety of yeasts. According to their chemical composition and specific functions in living systems, these molecules are classified as structural and enzymatic mannoproteins. The most abundant type of mannoprotein is structural, which consists of a small protein portion linked to a larger carbohydrate portion (mannopyranosyl), whereas enzymatic mannoproteins contain more protein moieties. Not only are these molecules effective emulsifiers, but they have also been linked to the stimulation of host immunity via the activation of immune cells and proteins as well as the induction of antibody production [24,25]. Figure 2 depicts the structure and mechanism of action of a number of significant emulsifiers produced by microorganisms through biotechnology processes.

## 3. Bioemulsifiers Derived from Microorganisms

Because of their unique properties relative to chemical surfactants, such as biodegradability, foaming, non-toxicity, efficiency, biocompatibility, at low concentrations, and high selectivity across a range of pH, temperatures, and salinities, bioemulsifiers are referred to as surface-active biomolecule materials [11]. Emulsifiers are abundant in nature and are produced by bacteria, fungi, and yeasts (Table 1).

On the other hand, marine microorganisms are a wealthy source of bioactive compounds, such as enzymes, biosurfactants, and drugs. Because of their unique interaction with cell membranes, biosurfactants have recently received interest in their antibacterial, anticancer, and antiviral properties. Due to the high cost of industrial manufacture, commercially accessible biosurfactants (such as sophorolipids, rhamnolipids and surfactin) are currently limited. As a result, innovative biosurfactants or alternative biosurfactant-producing strains are in high demand. The ability of marine *Bacillus* species to grow in high-salinity conditions has recently been described [26,27]. According to Liu et al. [28], three *Bacillus* species from the sea have been discovered to be able to use oil and perform emulsification.

## 4. Physicochemical Properties of Bioemulsifiers

The capacity of bioemulsifiers to stabilize emulsions by enhancing their kinetic stability has enhanced their application in the pharmaceutical, food and petroleum industries. Numerous investigations have been performed on bioemulsifiers, whose effective emulsifying action is dependent on their chemical composition [54,55]. According to Willumsen and Karlson [56], surfactants and emulsifiers are two types of surface-active biomolecules that are utilized for emulsions stabilization. Some biomolecules, on the other hand, have both surfactant and emulsifying capabilities, which contributes to their unique functions and wide range of industrial applications. Table 2 reports the physico-chemical properties of bioemulsifiers.

## 5. Characterization of Bioemulsifiers by Various Chromatographic and Spectroscopic Techniques

Various techniques such as chromatographic and spectroscopic methods were applied to fully characterize the structure of bioemulsifiers. A combination of these procedures is highly useful for compound characterization.

One of the most often used techniques for detecting bioemulsifiers is thin layer chromatography (TLC). Table 3 summarizes the various solvents used for the detection of different functional groups from bioemulsifiers produced by microorganisms using TLC method.

In gas chromatography-mass spectrometry (GC-MS), the sample must be hydrolytically cleaved between the carbohydrate or peptide/protein part of the bioemulsifiers and the lipid portions in order to be analyzed in a GC or GC-MS equipment. As a consequence, fatty acid chains are derivatized to fatty acid methyl esters (FAME) and then converted to trimethylsilyl (TMS) derivatives for GC or GC-MS analysis [34]. The diazomethane esterification is an important step for the detection of compounds using GC-MS. Bio-emulsion from oil degrading *R. erythropolis* 3 C-9 was characterized by Peng et al. [72]. The FA (fatty acid) was esterified from crude extracts with 2 mol/L HCl in methanol at 100 °C (40 min). The FAME were then recovered with hexane and concentrated to 1 mL for GC-MS analysis under nitrogen atmosphere. The temperature graduated and was kept between 60 and 260 °C at 5 °C/min. A one µL of sample was applied to the GC-MS analysis. The purified carbohydrate sample was prepared by removing the aqueous phase through freeze drying and then extracting with pyridine to remove all ions. After that, the pyridine was removed using the evaporation under vacuum at 40 °C. The saccharide part of the sample was dissolved in distilled water and utilized for further analysis.

In high-performance liquid chromatography (HPLC), the sample is analyzed in the chromatographic column thanks to the mobile phase pumped by plumping system. The detector responds to the elution of the sample, signaling a peak on the chromatogram [73]. Lipopeptide separation is commonly accomplished using HPLC coupled to refractive index, UV, fluorescence, electrochemical, near-infrared, MS, NMR, and light scattering [73,74]. The sample is treated with trifluoroacetic acid (TFA) and centrifuged to remove solid particles before being analyzed in an HPLC facility. In addition, if the HPLC is equipped with an MS or evaporative light scattering detectors (ELSD), glycolipids can also be separated and identified sequentially. The polarity of components is the main factor to identify the separated products and provide them in individual peaks to study the structure of each moiety. HPLC with MS detection is important to identify the molecular mass of each fraction.

Nuclear magnetic resonance (NMR) is based on magnetic moment changes in atoms when an external magnetic field is applied. A nucleus in a high magnetic field absorbs radio frequency radiation [75]. NMR can give direct information concerning the functional groups and the bond positions for the protein, lipid and carbohydrate molecules. NMR experiments can also possibly identify the location of each functional group and inform about the constitutional isomers. The most common solvents utilized are acetic acid, acetone, chloroform, dimethyl sulfoxide, benzene, and methanol pyridine. The samples are hydrolyzed using HCl; then, the FA is extracted and detected through NMR. The glycolipids should be dissolved in deuterated chloroform before performing a series of 1D (1H and 13C) and 2D (such as HMQC, ROSY, COSY, and HMBC) NMR investigations. The NMR approach was used to conduct detailed investigations of glycolipid, which was recently published in the literature [76,77].

Fourier-transform infrared spectroscopy (FT-IR) can identify unknown mixture components based on functional groups. Usually, 1 mg of freeze-dried, purified biosurfactant is ground with 100 mg of potassium bromide and pressed for 30 s to produce translucent pellets. The analysis uses an FT-IR device with a spectrum ranging from 400 to 4000 cm^−1^ [78,79]. Several studies used FT-IR for bioemulsifiers’ characterization; Gudiña et al. [80] studied the ability of a *Paenibacillus* sp. strain isolated from crude oil to produce the bioemulsifier. A preliminary chemical characterization by FT-IR, carbon and proton nuclear magnetic resonance (^13^C and ^1^H NMR) and size exclusion chromatography observed that the bioemulsifier is a low molecular weight oligosaccharide–lipid complex. In addition, there is an effective bio-surfactant-producer and hydrocarbon degrading bacterial strain, *Rhodococcus* sp. HL-6 was isolated from the Xinjiang oil field using diesel oil as a sole source of carbon. The produced biosurfactant (BS) characterization was made by thin-layer chromatography (TLC) and FT-IR [81,82].

Fast atom bombardment-mass spectrometry (FAB-MS), using a high-energy beam of xenon atoms and cesium ions, scatters the sample and matrix (m-nitro benzyl alcohol) from the probe’s surface. The biosurfactants are typically dissolved in methanol and mixed with matrix [83].

Electrospray ionization-mass spectrometry (ESI-MS) is a soft ionization technique utilized to produce gas-phase ions for high-molecular-weight biological molecules. Such a technique can be used with an HPLC (HPLC/ESI-MS) to gain a comprehensive understanding of the molecular structure [84].

The scanning electron microscopy (SEM) analysis was performed with the FEI QUANTA 200 FEG HR-SEM model at 8 mm working distance and 30 kV. On the sample holder, a very small amount of the specimen was placed, and thin layer of the samples were prepared on special carbon-coated paper. Using blotting paper, the excess solution was separated, and the SEM film was dried under a mercury lamp for five minutes [85].

The laser scanning confocal microscope (LSCM) is the most equipment using for studying the structure and stability of any emulsions [86]. In addition, LSCM is the best way to differentiate between the lipophilic and hydrophilic phases, the droplet size and distribution of oil bio-emulsion [87]. Various analytical methods namely, HPLC, IR, GC-MS and NMR, are used to characterize bioemulsifiers are listed in Table 4.

## 6. Applications of Bioemulsifiers in Food Industry

**Figure 3:**
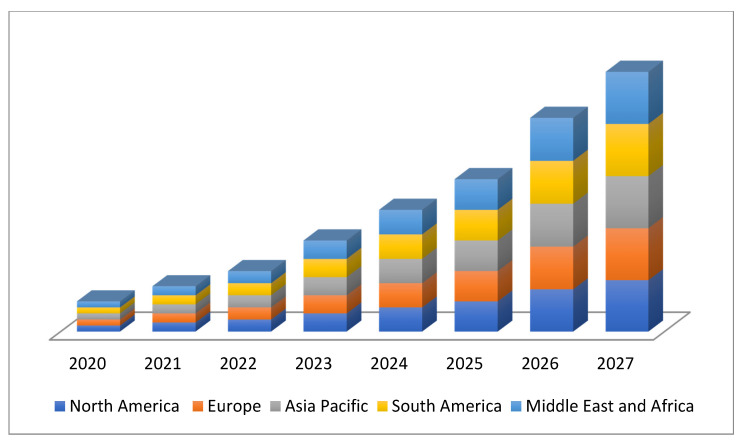
Worldwide emulsifiers market size.

The marketing of emulsifiers is expected to reach a value of USD 17.53 billion by 2027, while registering this growth at a rate of 6.90% for the forecast period of 2020 to 2027 [94]. Growing global demand for packaged foods worldwide is expected to create a new business opportunity for the market (Figure 3) [88]. The increasing use of emulsifiers in food products such as infant, child nutrition products and snacks are expected to enhance the market growth. Other factors such as increasing population health consciousness, rising disposable income, expansion in the cosmetics and personal care industry, and increasing concern about the food safety and quality will further provide the emulsifiers market in the forecast period of 2020 to 2027. However, these chemical emulsifiers cause negative impacts on gut health through impaired intestinal barrier function and increasing the incidence of inflammatory bowel disease (IBD). Researchers have produced emulsifiers using natural resources and the availability of a minor or non-toxic alternative, especially microorganisms due to restricted resources and high costs [95,96].

**Figure 4:**
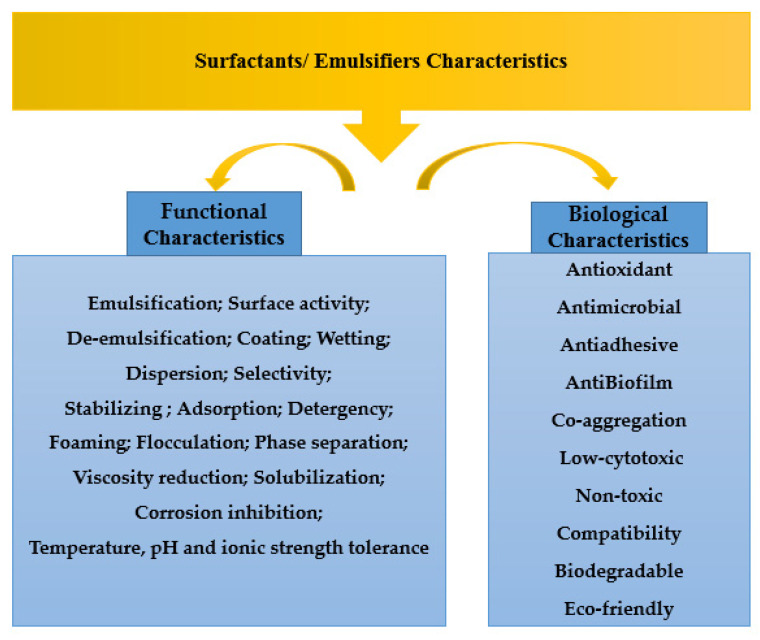
Various biological and functional properties of bioemulsifiers.

The unique natural properties of bioemulsifiers are the amphiphilicity (hydrophilic and hydrophobic) and their ability to reduce interfacial tension and surface area. Other interesting properties viz., coagulation, emulsification, cleansing, wetting, foaming ability, phase separation, surface activity and reduction in the oil viscosity permit their exploitation in many industries. Bioemulsifiers have a wide range of structural, compositional, and functional features due to the variety of their microbial origins, which include fungi [49,97], bacteria [98], and actinomycetes [99]. Figure 4 shows the main characteristics most bioemulsifiers may have to be considered as “emulsifier”. The bioemulsifiers such as liposan from Candida lipolytica were able to stabilize the emulsions of vegetable oils and water. It was also able to stabilize the corn oil, cottonseed oil, peanut oil, and soybean oil emulsions [100].

**Figure 5:**
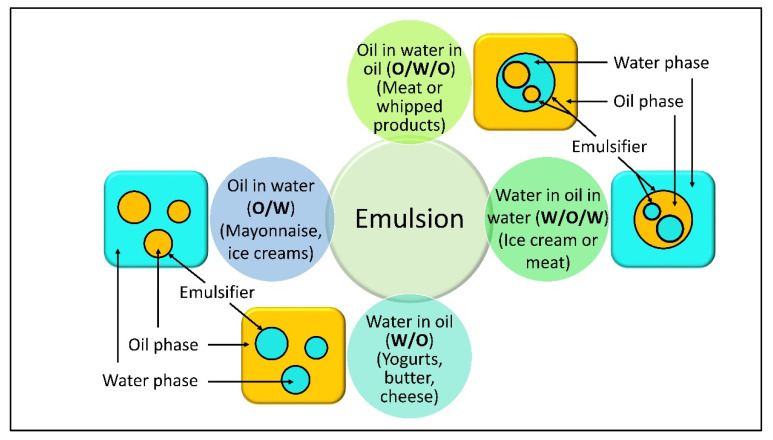
Three main forms of emulsions important in a variety of foods.

The formulation of food determines several phases among particles [101]. Figure 3 shows basically the main types of emulsions that are important in a variety of foods. This precise structural organization of bioemulsifier molecules allows surface-active agents/emulsifiers to quintessence at the O/W interphase, leading to boosting the modynamic stability of an unstable system [102]. Because of their amphiphilic nature, emulsifiers have significant emulsifying powers and may be molded with starches and protein fractions of food items. Additionally, the partly digested fatty components are adequately emulsified/homogenized by bioemulsifiers. The emulsifier binds to protein portions of food items, causing them to aggregate together [103]. Mannor protein producing Saccharomyces cerevisiae facilitates the stabilization of W/O emulsions for products such as mayonnaise and ice creams [104]. Water in oil in water (W/O/W) and oil in water in oil (O/W/O) are two more sophisticated types of duplex emulsions (multiple) (Figure 5).

Lipopolysaccharides, heteropolysaccharides, lipoproteins, glycoproteins, and proteins are regarded as beneficial for commercial applications as bioemulsifiers. A variety of new uses of new and well-known bioemulsifiers have been described in the recent three years. The excellent properties of both microbial produced biosurfactants and bioemulsifiers have features that make them desirable as natural emulsifiers for foods. Different studies have described the use of glycolipids to stabilize fat emulsions as well as glycolipids and lipopeptides as rheology modifiers in cookie and muffin dough [3,105]. Other studies have found that bioemulsifiers (such as exopolysaccharides and mannoproteins) have a high potential for aroma emulsification [106].

### Incorporation of Bioemulsifiers in Food Formulations

1. Salad dressing formulation was prepared using sunflower oil, vinegar, water, egg powder, sugar, salt, starch, etc. with Candida-derived bioemulsifier (*C. utilis* 0.2–0.8% (*w*/*v*) combined with guar gum/carboxymethyl cellulose. The consistency and texture was improved using 0.7% of bioemulsifier [107].
2. Muffins were prepared using Galactan Exopolysaccharide (EPS) 1% (*w*/*v*) along with vanillin and cardamom flavors. It showed a better texture, sensorial property, springiness, color and flavor stability than control [108].
3. Cookie dough formulation incorporated bioemulsifier from *S. cerevisiae* URM 6770, partially (2% (*w*/*v*)) or completely (4% (*w*/*v*)) substituting egg yolk in the existing formulation, and it showed similar physicochemical properties along with increasing the energy value of the cookies by providing fatty acids in the end product [3]. Table 5 summarizes some of the most interesting findings.

## 7. Conclusions

With the increasing trend toward natural substitutes for synthetic ones, bioemulsifiers have gained importance over time. This is due to the production from renewable resources, having better surface tension reducing or interfacial activity, low toxicity, better physicochemical properties and the emulsifying and stabilizing effects in the food industry. The obstacles in complete replacement by these biomolecules are lower yields, higher production costs, variations in the final properties which have led to lower commercial viability and the utilization of bioemulsifiers in the food industry. The cost-effective, large-scale production of bioemulsifiers and the study of interactions of bioemulsifiers with other ingredients in the food formulation needs further research and optimization to increase utilization on a greater scale to make bioemulsifiers a success. In spite of these difficulties, bioemulsifiers will continue to grow in the near future, hence proving to be a natural and safer alternative to its chemical counterparts.

## Figures and Tables

**Table 1:** Bioemulsifiers produced by bacteria, yeast and fungi.

*Bacteria* Sources			*Yeast* Sources			Fungi Sources		
Bacteria	Bioemulsifiers	References	Yeast	Bioemulsifiers	References	Fungi	Bioemulsifiers	References
*Pseudomonas fluorescens*	Viscosin	[29]	*Torulopsis petrophilum*	Sophorolipids	[30]	*Candida sphaerica UCP0995*	Sophorolipids	[31]
*Pseudomonas aeruginosa*	Rhamnolipids	[32]	*Torulopsis apicola*	Sophorolipids	[33]	*Candida lipolytica Y-917*	Sophorous lipid	[32]
*Pseudomonas fluorescens*	Carbohydrate-lipid complex	[32]	*Pseudozyma rugulosa*	Mannosylerythritol lipids	[34]	*Candida utilis*	NDA	[35]
*Bacillus amyloliquefaciens*	Surfactin/Iturin	[36]	*Pseudozyma aphidis*	Mannosylerythritol lipids	[37]	*Candida ingens*	Fatty acids	[38]
*Bacillus subtilis*	Subtilisin	[39]	*Kurtzmanomyces* sp.	Mannosylerythritol lipids	[40]	*Candida lipolytica*	Carbohydrate-protein-lipid	[41]
*Bacillus subtilis*	Lichenysin	[42]	*Kurtzmanomyces* sp. *I-11*	Mannosylerythritol lipids	[43]	*Candida tropicalis*	Liposan	[44]
*Bacillus licheniformis K51*	Peptide lipids	[45]	*Debaryomyces polymorphus*	Carbohydrate protein-lipid	[46]	*Candida bombicola*	Sophorolipids	[47]
*Bacillus pumilus A1*	Rhamnolipids	[48]	*Saccharomyces cerevisiae*	Mannoprotein	[49]	*Candida (torulopsis)*	Sophorolipids	[50]
*Bacillus* spp.	Hydrocarbon-lipid-protein	[51]	*Kluyveromyces marxianus*	Mannoprotein	[52]	*Candida lipolytica*	Carbohydrate-protein	[53]

**Table 2:** Physico-chemical properties of bioemulsifiers.

Bioemulsifiers Class	Microbial Origin	Physicochemical Properties	References
Glycoprotein	*Solibacillus silvestris* AM1	Pseudoplastic non-Newtonian rheological property	[57]
Alasan	*Acientobacter radioresistens KA53*	Emulsification and solubilization activity	[58]
Uronic acid bioemulsifiers	*Halomonaseurihalina Klebsiella* sp.	Emulsification properties	[59]
*Proteoglycan*	*Acinetobacter calcoaceticus MM5*	Emulsifies heating oils	[60]
*Lipo-heteropolysaccharides*	*Acinetobacter bouvetii UAM25*	Emulsifying polycyclic aromatic hydrocarbon	[61]
Lipoglycan	*Acinetobacter baumanii*	Emulsification of edible oils	[62]
Glycolipid	*Acinetobacter* sp.	Surface active agent	[63]
Glycolipid	*Acinetobacter* spp.	Stable emulsions only in the presence of edible oils	[64]
Amyloid	*Solibacillus silvestris* AM1	Strengthening cell surface interactions such as aggregation, biofilm formation and adhesion	[65]

**Table 3:** Characterization of bioemulsifiers produced by microorganisms using TLC techniques using various solvents systems.

Bioemulsifiers Type	Organism	Solvent System	Functional Groups	Reference
Glycolipid	*Pseudomonas* sp.	Chloroform; methanol; water 65:25:5	Glycolipid	[66]
Lipopeptide	*Bacillus subtilis*	Butanol; acetic acid; water 4:1:1 methanol; 6 N HCl; water; pyridine 60:3:19:5:15	Amino acids	[67]
Lipopeptides	*Enterobacter cloacae C3*	Chloroform/methanol/water (65:25:4).	lipopeptides	[68]
Glycolipids Ustilagic acid	*Ustilago maydis*	Chloroform; methanol; water 65:25:4	Sugar	[69]
Glycolipid	*Bacillus* sp.	Chloroform; methanol; acetic acid; water 25:15:4:2	Carbohydrate Lipid	[70]
Lipopeptide	*Bacillus subtilis*	Butanol; acetic acid; water 4:1:1 Methanol; 6 N HCl; water; pyridine 60:3:19:5:15	Amino acids	[71]

**Table 4:** Characterization of bioemulsifiers produced by different Microorganisms using various analytical methods.

Microorganism	Bioemulsifiers Type	HPLC	FT-IR	GC-MS	NMR	Reference
*Pseudomonas aeruginosa*	Rhamnolipid	+	−	−	−	Haba et al. [88]
*Pseudomonas putida*	Bioemulsifier	+	−	−	+	Bonilla et al. [89]
*Pseudomonas putida 21 BN*	Rhamnolipid	−	+	−	−	Tuleva et al. [90]
*Bacillus* sp.	Exopolysaacharide	−	−	−	−	Yun and Park [91]
*Bacillus licheniformis*	Lipopeptide	+	−	+	+	Yakimov et al. [92]
*Candid picola*	Glycolipid	−	−	+	−	Hommel et al. [93]
*Yarrowia lipolytica*	Yansan	−	+	+	−	Amaral et al. [13]

+: Test carried out by authors. −: Test not done by authors.

**Table 5:** The latest (2015–2022) findings on some bioemulsifiers exhibiting potential activity.

Bioemulsifiers	Microorganisms	Activity	Application	Reference
Lipopeptide	*Bacillus licheniformis MS48*	Improving textural and sensorial properties	Yogurt	[109]
Glycolipoprotein	*Acinetobacter indicus M6*	Antibacterial	Food control	[110]
Proteoglycan	*Meyerozyma caribbica*	Emulsifiers	Food industry	[111]
Exopolysaccharides (EPS)	*Rhodobacter johrii CDR-SL 7 Cii*	Emulsifier Emulsion Stabilizer	Food industry	[112]
Carbohydrate–lipid–protein complex	*Candida utilis*	Emulsifiers	Corn oil and Sunflower oil	[108]
Succinoglycan exopolysaccharide	*Rhizobium radiobacter CAS*	emulsion stabilization	Soybean oil	[113]
EPS	*Pseudomonas fluorescens*	Emulsifier	Food industry	[114]
EPS	*Chromohalobacter canadensis 28*	Emulsifier Emulsion Stabilizer Foamer	Food industry	[108]
Glycoprotein	*Lactobacillus plantarum* subsp.	Emulsifiers	Food industry	[115]
Lipopeptide	*Nesterenkonia* sp. *MSA31*	Antioxidant, Emulsifier, Emulsion Stabilizer	Food industry	[106]
emulsan-alginate	*Pseudomonas stutzeri 273*	Removing protein-based toxins from food products	Food-processing contamination	[116]
Polyketide derivative	*Penicillium chrysogenum*	Emulsifiers	Oil	[117]

## Data Availability

Not applicable.
